# Occlusion and catheter ablation using a large-size cryoballoon for various pulmonary veins: a case series

**DOI:** 10.1093/ehjcr/ytad593

**Published:** 2023-11-24

**Authors:** Reisuke Yoshizawa, Hiroki Sasaki, Takashi Urushikubo, Yohei Sawa, Shingen Owada

**Affiliations:** Division of Cardiology, Department of Internal Medicine, Iwate Medical University School of Medicine, Shiwa, Japan; Division of Cardiology, Department of Internal Medicine, Iwate Medical University School of Medicine, Shiwa, Japan; Division of Cardiology, Department of Internal Medicine, Iwate Medical University School of Medicine, Shiwa, Japan; Division of Cardiology, Department of Internal Medicine, Iwate Medical University School of Medicine, Shiwa, Japan; Division of Cardiology, Department of Internal Medicine, Iwate Medical University School of Medicine, Shiwa, Japan

**Keywords:** Cryoballoon, Catheter ablation, Atrial fibrillation, Pulmonary vein isolation, Occlusion, Case report

## Abstract

**Background:**

It is established that pulmonary vein isolation using the POLARx™ (Boston Scientific, Marlborough, MA, USA) cryoballoon is a rapid, safe, and effective approach. The new POLARx™ FIT (Boston Scientific), which is expandable from 28 to 31 mm in diameter, is currently available. However, there is limited evidence available regarding the treatment of atrial fibrillation in this setting. In this article, we report a case series of cryoballoon ablation in patients with atrial fibrillation using POLARx™ FIT.

**Case summary:**

This case series describes a comparison of obstruction in three patients with pulmonary veins of different shapes and diameters undergoing cryoballoon ablation and pulmonary vein isolation with a 31 mm diameter balloon.

**Discussion:**

Cryoballoon ablation using the 31 mm mode of POLARx™ FIT has the potential to provide safe and stable pulmonary vein isolation with good occlusion for a variety of pulmonary vein geometries. In this case series, the 31 mm mode of the POLARx™ FIT resulted in better pulmonary vein occlusion than the 28 mm mode in patients with large left atria and large pulmonary veins, including the left common pulmonary vein. This approach may be considered a first-line therapy option of cryoballoon ablation in patients with atrial fibrillation.

**Learning points**

- The 31 mm POLARx™ FIT has the potential to provide good occlusion for pulmonary veins of various shapes.
- Cryoballoon ablation using the 31 mm mode of POLARx™ FIT for the treatment of atrial fibrillation allows safe and reliable pulmonary vein isolation with fewer freezing cycles for pulmonary veins of various shapes.

## Introduction

Cryoballoon ablation (CBA) is widely used for the treatment of atrial fibrillation (AF).^1^ Pulmonary vein isolation (PVI) using the POLARx™ (Boston Scientific, Marlborough, MA, USA) cryoballoon is a rapid, safe, and effective approach.^2^ Better occlusion of the pulmonary vein (PV) by the balloon is associated with lower minimum attainable temperatures, as well as better success rates of PVI and long-term results in terms of isolation efficacy.^3–6^ However, it is difficult to occlude some PVs due to the anatomy of the left atrium or PV.^7^ The new expandable POLARx™ FIT (Boston Scientific) (diameter: 28–31 mm) is currently available. However, therapeutic outcomes are not well documented. Herein, we describe the first application of CBA using POLARx™ FIT for the treatment of AF in patients with various PV anatomies.

## Summary figure

**Figure:**
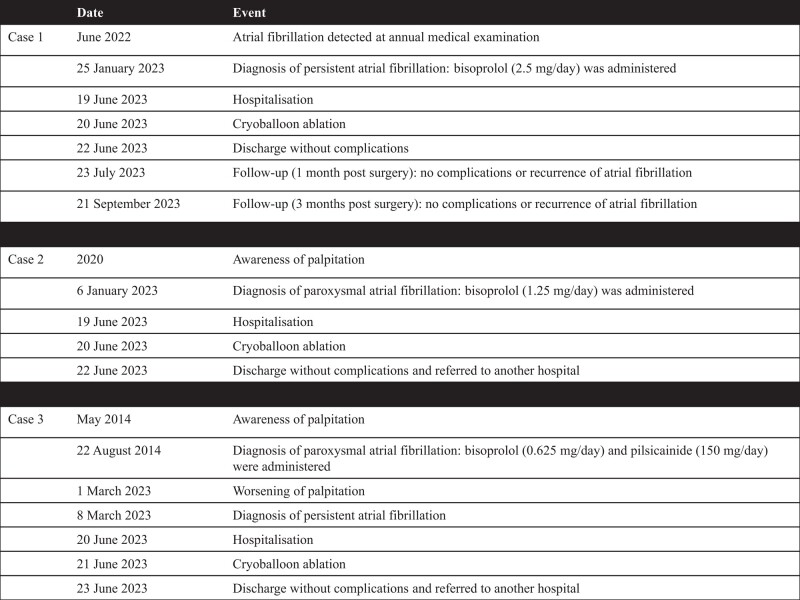


## Anatomic parameters obtained through pre-operative multidetector computed tomography

All patients underwent multidetector computed tomography (CT) before catheter ablation to obtain the contour of the left atrial cavity. The four major PVs were identified, namely the left superior pulmonary vein (LSPV), left inferior pulmonary vein (LIPV), right superior pulmonary vein (RSPV), and right inferior pulmonary vein (RIPV). The following anatomic parameters were measured through volume rendering: major and minor diameters of the PV ostium; length of the PV trunk from the ostium to the bifurcation (PVTL); and thickness of the left lateral ridge (LLRT) between the left PVs and left atrial appendage.^8^ The ovality index of the PV ostia was also calculated using the following formula: 2 × (major diameter − minor diameter)/(major diameter + minor diameter).^9^ The PV ostium was defined as the point of inflection between the left atrial wall and PV wall.^10^

## Catheter ablation procedure

Patients underwent catheter ablation under conscious sedation or general anaesthesia depending on patient/operator preference. All procedures were performed with uninterrupted anticoagulation therapy and intravenous heparin administration to achieve an activated clotting time > 300 s throughout the procedure. Transseptal punctures were performed through the SL0 sheath (Abbott, Abbott Park, IL, USA) using a RF-needle (Boston Scientific) and a guidewire. Subsequently, the sheath was exchanged for a 15 F steerable sheath (POLARSHEATH™; Boston Scientific). A 20 mm circular mapping catheter (POLARMAP™; Boston Scientific) and cryoballoon catheter (POLARx™ FIT; Boston Scientific) were advanced into the left atrium via POLARSHEATH™. The PV potentials and spontaneous firing from all PVs were confirmed using POLARMAP™ at the initiation of cryoablation. For all PVs, before the cryoablation, the seal between the 28 mm and 31 mm modes and venous ostium was assessed with an injection of 50% diluted contrast medium to compare the occlusion grade^11^: grade 4: complete occlusion; grade 3: minimal leakage; grade 2: moderate leakage; and grade 1: severe leakage. Following the detection of occlusion, cryothermal energy was initiated using only the 31 mm mode of POLARx™ FIT; the LSPV was treated for 4 min, while the other PVs were treated for 3 min. During ablation, if the PV potentials were visible during the delivery of energy, the time-to-isolation (TTI) was recorded.^3,12–14^ Time-to-isolation was defined as the period of time required to achieve a PVI by monitoring the real-time PV potentials on the POLARMAP™. To avoid phrenic nerve injury, all cryoballoon applications were performed under diaphragmatic electromyography monitoring.^12^ We also collected data on procedural characteristics, including the number of cryoapplications, total cryoballoon application time, TTI, and nadir balloon temperature. The time interval required to reach −30°C (FT−30) and −40°C (FT−40) from the initiation of freezing was determined. Moreover, the time interval required to reach 0°C (TT0) and 20°C (TT20) from the initiation of thawing was measured. The interval thaw time at 20°C was selected since this is generally the temperature limit at which the balloon is manually stretched by the operator on termination of the cryoballoon application.

## Case 1

**Figure 1:**
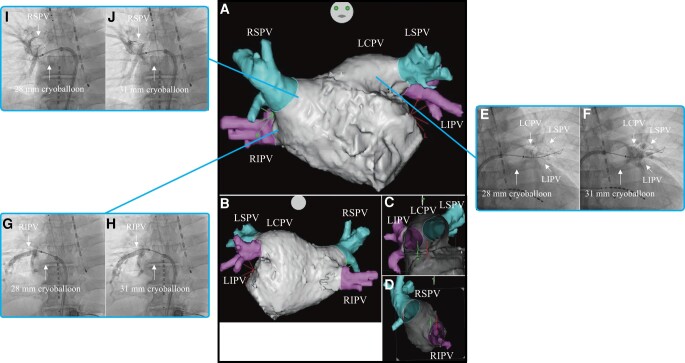
Three-dimensional images obtained through a multi-slice CT scan of Case 1 in the anteroposterior (*A*), posteroanterior (*B*), right-lateral (*C*), and left-lateral (*D*) views. Venogram of the LCPV during cryoballoon ablation using the 28 mm (*E*) and 31 mm (*F*) modes. Venogram of the RIPV during cryoballoon ablation using the 28 mm (*G*) and 31 mm (*H*) modes. Venogram of the RSPV during cryoballoon ablation using the 28 mm (*I*) and 31 mm (*J*) modes. CT, computed tomography; LCPV, left common pulmonary vein; RIPV, right inferior pulmonary vein; RSPV, right superior pulmonary vein.

A 60-year-old male was diagnosed with AF during an annual medical examination in 2022. He had a history of diabetes and had undergone catheter ablation for premature ventricular contractions 22 years ago. He was diagnosed with persistent AF in 2023 and received treatment with bisoprolol (2.5 mg/day). However, AF was refractory to the treatment. He was referred for catheter ablation. Examination showed that he was haemodynamically stable. Physical examination, chest X-ray examination, and echocardiography did not yield evidence of clinically overt structural and/or organic heart disease. Three-dimensional images obtained from a multi-slice CT scan are shown in *Figure 1A*–*D*.

Firstly, PVI was performed on the left common pulmonary vein (LCPV). On venography before freezing, the occlusion grade was grades 2 and 4 for the 28 mm and 31 mm modes, respectively, and both LSPV and LIPV were depicted (*Figure 1E* and *F*). The nadir balloon temperature was −65°C. Total cryoballoon application time was 223 s, while TTI was 48 s. FT−30 was 28 s, FT−40 was 29 s, TT0 was 41 s, and TT20 was 75 s. Secondly, PVI was performed on the RIPV. On venography before freezing, the occlusion grade was grade 4 for both the 28 mm and 31 mm modes (*Figure 1G*and*H*). The nadir balloon temperature was −64°C. Total cryoballoon application time was 180 s and TTI was 24 s. FT−30 was 26 s, FT−40 was 28 s, TT0 was 27 s, and TT20 was 76 s. Finally, PVI was performed on the RSPV. On venography before freezing, the occlusion grade was grade 4 for both 28 mm and 31 mm modes. The occlusion for the 31 mm mode was noted at a more proximal position than that for the 28 mm mode (*Figure 1I*and*J*). The nadir balloon temperature was −65°C. Total cryoballoon application time was 170 s and TTI was 21 s. FT−30 was 26 s, FT−40 was 29 s, TT0 was 31 s, and TT20 was 124 s. The patient was discharged from hospital 2 days after the catheter ablation. Three months after catheter ablation, he had an uneventful course without evidence of recurrence.

## Case 2

**Figure 2:**
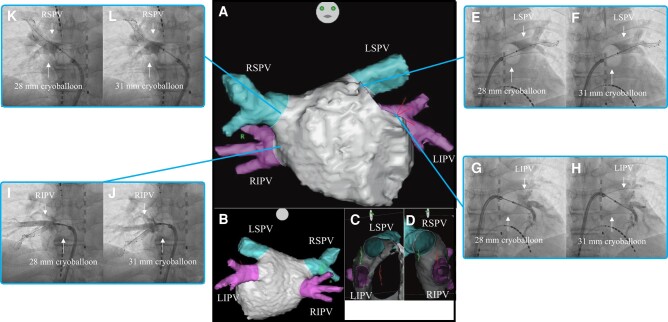
Three-dimensional images obtained through a multi-slice CT scan of Case 2 in the anteroposterior (*A*), posteroanterior (*B*), right-lateral (*C*), and left-lateral (*D*) views. Venogram of the LSPV during cryoballoon ablation using the 28 mm (*E*) and 31 mm (*F*) modes. Venogram of the LIPV during cryoballoon ablation using the 28 mm (*G*) and 31 mm (*H*) modes. Venogram of the RIPV during cryoballoon ablation using the 28 mm (*I*) and 31 mm (*J*) modes. Venogram of the RSPV during cryoballoon ablation using the 28 mm (*K*) and 31 mm (*L*) modes. CT, computed tomography; LIPV, left inferior pulmonary vein; LSPV, left superior pulmonary vein; RIPV, right inferior pulmonary vein; RSPV, right superior pulmonary vein.

A 64-year-old male without medical history was diagnosed with paroxysmal AF in 2023 and received treatment with bisoprolol (1.25 mg/day). However, AF was refractory to the treatment. He was referred for catheter ablation. Examination showed that he was haemodynamically stable. Physical examination, chest X-ray examination, and echocardiography did not yield evidence of clinically overt structural and/or organic heart disease. Three-dimensional images obtained through a multi-slice CT scan are shown in *Figure 2A*–*D*.

Firstly, PVI was performed on the LSPV. On venography before freezing, the occlusion grade was grade 4 for both the 28 mm and 31 mm modes (*Figure 2E*and*F*). The nadir balloon temperature was −57°C. Total cryoballoon application time was 240 s and TTI was 36 s. FT−30 was 28 s, FT−40 was 32 s, TT0 was 19 s, and TT20 was 55 s. Secondly, PVI was performed on the LIPV. On venography before freezing, the occlusion grade was grade 4 for both the 28 mm and 31 mm modes (*Figure 2G*and*H*). The nadir balloon temperature was −54°C. Total cryoballoon application time was 180 s and TTI was 15 s. FT−30 was 27 s, FT−40 was 29 s, TT0 was 15 s, and TT20 was 35 s. Thirdly, PVI was performed on the RIPV. On venography before freezing, the occlusion grade was grade 4 for both the 28 mm and 31 mm modes (*Figure 2I*and*J*). The nadir balloon temperature was −57°C. Total cryoballoon application time was 180 s and TTI was 26 s. FT−30 was 26 s, FT−40 was 30 s, TT0 was 16 s, and TT20 was 56 s. Finally, PVI was performed on the RSPV. On venography before freezing, the occlusion grade was grade 4 for both the 28 mm and 31 mm modes (*Figure 2K*and*L*). The nadir balloon temperature was −62°C. Total cryoballoon application time was 180 s and TTI was 21 s. FT−30 was 26 s, FT−40 was 28 s, TT0 was 24 s, and TT20 was 92 s. The patient was discharged from hospital 2 days after the catheter ablation and referred to another hospital. Four months after catheter ablation, he had an uneventful course without evidence of recurrence.

## Case 3

**Figure 3:**
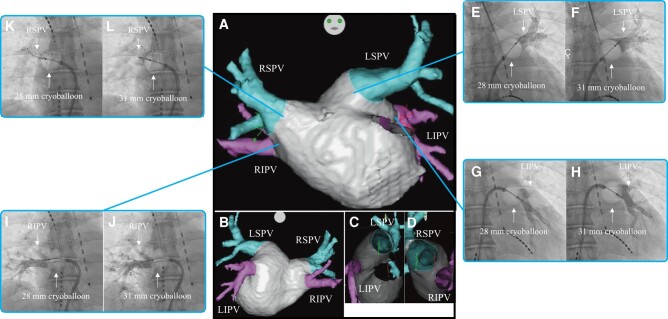
Three-dimensional images obtained through a multi-slice CT scan of Case 3 in the anteroposterior (*A*), posteroanterior (*B*), right-lateral (*C*), and left-lateral (*D*) views. Venogram of the LSPV during cryoballoon ablation using the 28 mm (*E*) and 31 mm (*F*) modes. Venogram of the LIPV during cryoballoon ablation using the 28 mm (*G*) and 31 mm (*H*) modes. Venogram of the RIPV during cryoballoon ablation using the 28 mm (*I*) and 31 mm (*J*) modes. Venogram of the RSPV during cryoballoon ablation using the 28 mm (*K*) and 31 mm (*L*) modes. CT, computed tomography; LIPV, left inferior pulmonary vein; LSPV, left superior pulmonary vein; RIPV, right inferior pulmonary vein; RSPV, right superior pulmonary vein.

A 65-year-old male was diagnosed with paroxysmal AF in 2014. He had history of hypertension. Bisoprolol (0.625 mg/day) and pilsicainide (150 mg/day) were administered. In 2023, his AF became persistent, and drugs were ineffective; hence, he was referred for catheter ablation. Examination showed that he was haemodynamically stable. Physical examination, chest X-ray examination, and echocardiography did not yield evidence of clinically overt structural and/or organic heart disease. Three-dimensional images obtained through a multi-slice CT scan are presented in *Figure 3A*–*D*.

Firstly, PVI was performed on the LSPV. On venography before freezing, the occlusion grade was grade 4 for both the 28 mm and 31 mm modes. The occlusion for the 31 mm mode was noted at a more proximal position than that for the 28 mm mode (*Figure 3E*and*F*). The nadir balloon temperature was −60°C. Total cryoablation application time was 240 s, and TTI was 32 s. FT−30 was 26 s, FT−40 was 28 s, TT0 was 20 s, and TT20 was 41 s. Secondly, PVI was performed on the LIPV. On venography before freezing, the occlusion grade was grade 4 for both the 28 mm and 31 mm modes. The occlusion for the 31 mm mode was noted at a more proximal position than that for the 28 mm mode (*Figure 3G*and*H*). The nadir balloon temperature was −50°C. Total cryoballoon application time was 180 s and TTI was 28 s. FT−30 was 27 s, FT−40 was 32 s, TT0 was 15 s, and TT20 was 38 s. Thirdly, PVI was performed on the RIPV. On venography before freezing, the occlusion grade was grade 4 for both the 28 mm and 31 mm modes. The occlusion for the 31 mm mode was noted at a more proximal position than that for the 28 mm mode (*Figure 3I*and*J*). The nadir balloon temperature was −56°C. Total cryoballoon application time was 180 s and TTI was 21 s. FT−30 was 26 s, FT−40 was 30 s, TT0 was 17 s, and TT20 was 47 s. Finally, PVI was performed on the RSPV. On venography before freezing, the occlusion grade was grades 1 and 3 for the 28 mm and 31 mm modes, respectively (*Figure 3K*and*L*). The nadir balloon temperature was −52°C. Total cryoballoon application time was 180 s and TTI was 21 s. FT−30 was 26 s, FT−40 was 29 s, TT0 was 11 s, and TT20 was 32 s. The patient was discharged from hospital 2 days after the catheter ablation and referred to another hospital. Four months after catheter ablation, he had an uneventful course without evidence of recurrence.

## Discussion

Clinical characteristics, echocardiographic data, and CT parameters are presented in *Tables 1* and *2*. Pulmonary vein isolation was achieved with a single freezing cycle, while pacing manoeuvres confirmed complete electrical isolation of all PVs. Peri- and post-procedural complications were ruled out, and the patients were discharged on the second post-interventional day.

**Table 1:** Patient demographics and clinical characteristics

	Case 1	Case 2	Case 3
Demographics			
Age, years	60	64	65
Height, cm	169.5	166	165.2
Body mass, kg	71.1	63.7	62.9
Body mass index, kg/m^2^	1.82	1.71	1.69
Comorbidity	Diabetes mellitus	Not available	Hypertension
Echocardiographic data			
Left atrial diameter, mm	44	37	46
LAD index, mm/m^2^	24.2	21.6	27.2
Left atrial volume, ml	79.5	56.6	119.8
LAV index, mL/m^2^	43.7	33.1	70.9
LV diastolic diameter, mm	53	49	62
LV ejection fraction, %	60	65	42
MR of ASE grade ≥ II	Not available	Not available	Not available
Medication			
Pre-procedural BB	Available	Available	Available
Pre-procedural AAD	Not available	Not available	Available

AAD, antiarrhythmic drug; ASE, American Society of Echocardiography; BB, beta-blockers; LAD, left atrial diameter; LAV, left atrial volume; LV, left ventricular; MR, mitral regurgitation.

**Table 2:** Computed tomography parameter, occlusion grade by venogram before freezing, and procedural and biophysical parameters

	Case 1	Case 2	Case 3
*CT parameters*			
LSPV			
Maximal diameter, mm	Not available	13.6	24.5
Minimal diameter, mm	Not available	11.2	22.6
Ovality index	Not available	0.19	0.08
LIPV			
Maximal diameter, mm	Not available	15	10.2
Minimal diameter, mm	Not available	6.8	9.3
Ovality index	Not available	0.75	0.09
RSPV			
Maximal diameter, mm	18.6	13.5	26.7
Minimal diameter, mm	15.7	10.7	18
Ovality index	0.17	0.23	0.39
RIPV			
Maximal diameter, mm	17.2	14.3	13.2
Minimal diameter, mm	12.3	10.9	12.1
Ovality index	0.33	0.27	0.09
LCPV			
Maximal diameter, mm	28.9	Not available	Not available
Minimal diameter, mm	17.7	Not available	Not available
Ovality index	0.48		
PVTL			
LSPV	Not available	26.4	28.7
LIPV	Not available	21.8	11.6
RSPV	13	25.5	4.6
RIPV	4.5	16.8	8
LCPV	26.5	Not available	Not available
LLRT			
LSPV	Not available	5.5	4.6
LIPV	Not available	5.2	4.1
LCPV	4.6	Not available	Not available
*Occlusion grade*			
28 mm mode			
LSPV	Not available	4	4
LIPV	Not available	4	4
RSPV	4	4	1
RIPV	4	4	4
LCPV	2	Not available	Not available
31 mm mode			
LSPV	Not available	4	4
LIPV	Not available	4	4
RSPV	4	4	3
RIPV	4	4	4
LCPV	4	Not available	Not available
*Procedural and biophysical parameters*			
LSPV			
Number of cryoapplications	Not available	1	1
Total cryoballoon application time (s)	Not available	240	240
TTI (s)	Not available	36	32
Nadir balloon temperature (°C)	Not available	−57	−60
FT−30 (s)	Not available	28	26
FT−40 (s)	Not available	32	28
TT0 (s)	Not available	19	20
TT20 (s)	Not available	55	41
LIPV			
Number of cryoapplications	Not available	1	1
Total cryoballoon application time (s)	Not available	180	180
TTI (s)	Not available	15	28
Nadir balloon temperature (°C)	Not available	−54	−50
FT−30 (s)	Not available	27	27
FT−40 (s)	Not available	29	32
TT0 (s)	Not available	15	15
TT20 (s)	Not available	35	38
RSPV			
Number of cryoapplications	1	1	1
Total cryoballoon application time (s)	170	180	180
TTI (s)	21	21	21
Nadir balloon temperature (°C)	−65	−62	−52
FT−30 (s)	26	26	26
FT−40 (s)	29	28	29
TT0 (s)	31	24	11
TT20 (s)	124	92	32
RIPV			
Number of cryoapplications	1	1	1
Total cryoballoon application time (s)	180	180	180
TTI (s)	24	26	21
Nadir balloon temperature (°C)	−64	−57	−56
FT−30 (s)	26	26	26
FT−40 (s)	28	30	30
TT0 (s)	27	16	17
TT20 (s)	76	56	47
LCPV			
Number of cryoapplications	1	Not available	Not available
Total cryoballoon application time (s)	223	Not available	Not available
TTI (s)	48	Not available	Not available
Nadir balloon temperature (°C)	−65	Not available	Not available
FT−30 (s)	28	Not available	Not available
FT−40 (s)	29	Not available	Not available
TT0 (s)	41	Not available	Not available
TT20 (s)	75	Not available	Not available

CT, computed tomography; FT−30, freezing time to −30°C; FT−40, freezing time to −40°C; LCPV, left common pulmonary vein; LIPV, left inferior pulmonary vein; LLRT, thickness of the left lateral ridge; LSPV, left superior pulmonary vein; PVTL, length of the PV trunk from the ostium to the bifurcation; RIPV, right inferior pulmonary vein; RSPV, right superior pulmonary vein; TTI, time-to-interval; TT0, thawing time to 0°C; TT20, thawing time to 20°C.

Firstly, good occlusion was obtained for PVs of various shapes using the 31 mm mode of POLARx™ FIT. Effective PV occlusion by cryoballoon was associated with lower minimum temperature, as well as better success rate of PVI and the long-term outcome in terms of the isolation effect.^3–6,11^ Due to the anatomical shape of the left atrium and PV ostia, some PVs cannot be completely occluded by cryoballoon.^7^ The 31 mm mode of the POLARx™ FIT used in this case series showed better PV occlusion than the 28 mm mode in cases with large left atria and large PVs (e.g. Case 1 and the superior PVs of Case 3), including LCPV. Furthermore, the 31 mm mode of POLARx™ FIT provided comparable occlusion for narrow PVs (e.g. Case 2 and inferior PVs of Case 3) with that of the 28 mm mode.

Secondly, PVI using the 31 mm mode of POLARx™ FIT was safe and reliable, and linked to fewer freezing cycles. Owing to the improved freezing effect of the cryoballoon, the majority of the PVs could be isolated in a single freezing cycle.^15^ Achieving acute PVI with a single shot in the left atrium and PV with large anatomical variations is difficult.^8^ In this case series, using the 31 mm mode of the POLARx™ FIT, it was possible to achieve PVI through a single freezing cycle, even in the large left atrium and PVs. In cryoablation for RIPV, early bifurcation prevents optimal cryoballoon alignment, and the frozen surface tends to float away from the PV ostia.^8,9^ In the present series, using the 31 mm mode of POLARx™ FIT in a similar case, it was possible to conduct PVI with a single freezing cycle. In the left PV, PVI may be difficult when there is a thinner LLRT and continuous sharp ridges of the left appendage due to the low contact area between the cryoballoon surface and the myocardium. In addition, a longer PVTL may complicate cryoballoon contact as the balloon is pushed into a more horizontal position.^8,9^ As shown in the case series, the 31 mm mode of the POLARx™ FIT allowed PV occlusion in a more proximal position compared with the 28 mm mode. This is because as the balloon expands from 28 to 31 mm, the intra-balloon pressure also increases from 2.5 psig to 7.5 psig, thereby reducing the risk of balloon extension into the PV. This may further reduce the risk of phrenic nerve injury during freezing and PV stenosis in the PVs. Furthermore, the PVI may be expanded to 31 mm to include the antrum area and carina compared with the 28 mm mode.

Additionally, ganglionated plexi (GP), which are modified by the effects of the cryoballoon,^16^ may be more extensively modified due to an increase in the size of the balloon. The benefits of adding GP ablation are well established.^17^ Notably, it has been reported that pulsed field ablation (a new single-shot device with equivalent efficacy to that of the cryoballoon)^18^ does not modify GP.^19^ Large-scale clinical trials are warranted to determine the long-term effect of the technique. Nevertheless, POLARx™ FIT has shown potential for effective PVI. Furthermore, pulsed field ablation has been linked to the development of cardiac tamponade.^18^ In contrast, the cryoballoon is associated with a low risk of cardiac tamponade owing to its soft balloon and circular mapping catheter. This evidence indicates that the cryoballoon allows for safe mapping and navigation.^20^ Of note, while specific company products are featured in this case series, there are no sponsorship or other conflicts of interest to declare.

In conclusion, the 31 mm mode of POLARx™ FIT showed potential for achieving good occlusion and PVI for PVs of various shapes. This case series suggests a promising application for large cryoballoons. However, larger clinical trials are warranted to further investigate the feasibility, effectiveness, and safety of this approach for PVs with more diverse anatomical features (e.g. common inferior pulmonary trunk and accessory PVs such as the right middle PV or LA roof vein).

## Data Availability

No new data were created or analysed in this article.

## Lead author biography

Reisuke Yoshizawa, MD, is an interventional cardiac electrophysiologist, and Assistant Professor, Iwate Medical University, Shiwa, Japan. He was in Teikyo University School of Medicine from 2002 to 2008, Iwate Prefectural Hanamaki Kosei Hospital, Hanamaki, Japan from 2008 to 2009, Iwate Prefectural Chubu Hospital, Kitakami, Japan from 2009 to 2013, Iwate Medical University, Morioka, Japan from 2013 to 2016, Assistant Professor, University of Tsukuba, Tsukuba, Japan from 2016 to 2017, Clinical Research Fellow in Cardiology, Iwate Prefectural Kamaishi Hospital, Kamaishi, Japan from 2017 to 2019, Chief in Cardiology. Currently, he works in Iwate Medical University, Shiwa, Japan since 2019.
